# Antimicrobial stewardship program in a pediatric intensive care unit of a tertiary care children’s hospital in Saudi Arabia – a pilot study

**DOI:** 10.1186/2047-2994-4-S1-P173

**Published:** 2015-06-16

**Authors:** LS AlAwdah, D AlShahrani, M AlShehri, T AlFawaz, N ElSidig, A AlAwfi, K Baba, A AlAqeel, A AlSharif, I AlHarfi, S Amjad, A AlDarwish, S Rasheed

**Affiliations:** 1 Pediatric Infectious Diseases, Children’s Specialized Hospital, King Fahad Medical City, Saudi Arabia; 2 Pediatric Infectious Diseases, Children’s Specialized Hospital, King Fahad Medical City, Saudi Arabia; 3 Microbiology Laboratory, King Fahad Medical City, Saudi Arabia; 4 Pediatric Intensive Care Unit, Children’s Specialized Hospital, King Fahad Medical City, Saudi Arabia; 5 Clinical Pharmacy Department, King Fahad Medical City, Saudi Arabia; 6 Quality Management Department, King Fahad Medical City, Saudi Arabia

## Introduction

Antimicrobial resistance is a serious global threat that needs urgent attention. Antimicrobial stewardship (AS) programs were proven to be effective in reducing antibiotic days in adults. Pediatric-specific data about the effectiveness of implementation of such programs are limited especially in the Middle East.

## Objectives

Our primary objective was to identify the top three antibiotics and measure their specific days of therapy (DOT) per 1000 pediatric intensive care unit (PICU) days per quarter before and after the AS intervention.

## Methods

We conducted a pilot prospective quality-improvement interventional study in a 20-bed PICU in a specialized tertiary care children’s hospital in Riyadh, Saudi Arabia from April to December 2014. Data about antimicrobial indication and utilization were gathered from study-designed forms. An AS member from pediatric infectious disease team rounded three times per week on all patients in PICU and provided antibiotic-related recommendations based on available bedside information and ensured adherence to antimicrobial restriction policy. Intervention also included educational sessions to physicians and nurses about proper use of antibiotics.

## Results

During the study period, 648 children out of 898 PICU admissions (72.2%) were utilizing antimicrobials. Vancomycin, Pipracillin/Tazobactam and Meropenum represented approximately 60% of the 36 antimicrobials used. Per quarter, antibiotic-specific DOT dropped from 348.5 to 320.5 (8.0%, p-value <0.001) for Vancomycin, 356.2 to 294.7 (17.3%, p-value <0.001) for Pipracillin/Tazobactam, 162.0 to 111.1 (31.4%, p-value <0.001) for Meropenum.

## Conclusion

Education and bedside real-time AS recommendations significantly dropped the use of Meropenum, Pipracillin/Tazobactam and Vancomycin. Moreover, AS program proved to be effective and promising in reducing antibiotic days in PICU.

## Disclosure of interest

None declared.

